# Tracing the historical foundations of infliximab in Crohn’s disease treatment: a cited reference analysis

**DOI:** 10.3389/fphar.2024.1498464

**Published:** 2024-11-13

**Authors:** Andy Wai Kan Yeung

**Affiliations:** Oral and Maxillofacial Radiology, Applied Oral Sciences and Community Dental Care, Faculty of Dentistry, The University of Hong Kong, Hong Kong, Hong Kong SAR, China

**Keywords:** Crohn’s disease, infliximab, cited reference analysis, CDAI, ACCENT I

## Abstract

**Introduction:**

The use of infliximab to treat Crohn’s disease patients has been evaluated for decades. The current work aimed to identify the historical roots of this research topic.

**Methods:**

The literature database Web of Science Core Collection was searched to identify relevant papers. Cited reference analysis on the identified literature set was performed using CRExplorer, a dedicated bibliometric software. The disruption index was computed with an automated routine described by Leydesdorff and Bornmann, which is freely available online. Based on data from citation count and reference list, the disruption index can range from −1 to +1, with −1 meaning a continuity from existing research and +1 meaning a disruption.

**Results:**

This analysis successfully identified key references dealing with infliximab use on Crohn’s disease patients, such as the original report that introduced the Crohn’s Disease Activity Index (CDAI) in 1976, the first case series reporting a favourable outcome of infliximab infusion on 10 patients published in 1995, the first randomized controlled trial published in 1997, the ACCENT I and ACCENT II trials published in 1999 and 2002, and a couple of European consensus guidelines on the diagnosis and management of Crohn’s disease.

**Conclusion:**

Cited reference analysis could reveal the historical origins of the use of infliximab in treating Crohn’s disease. Highly cited references included CDAI, important early clinical studies, and European consensus guidelines. The important cited references identified by the analysis provided solid foundation to support subsequent research.

## 1 Introduction

Crohn’s disease is a type of inflammatory bowel diseases (IBD) and has affected many people around the world. The collective data during the period of 1990–2016 have indicated that the prevalence of Crohn’s disease is higher among European and North American countries, such as the United States (96.3 per 100,000), but lower in Asian countries, such as Japan (18.6 per 100,000) (Ng et al., 2017). Crohn’s disease is a debilitating disease that is chronic inflammatory in nature, progressively damaging the gastrointestinal tract with periods of relapses and remission; and developing strictures, fistulas, and abscesses that often require surgical management (Torres et al., 2017). Recent papers from medicine (Caprilli et al., 2002; Sands, 2007; Hindryckx et al., 2014) have acknowledged Targan et al. (1997) as the first randomized controlled trial of infliximab on treating Crohn’s disease patients. To the best understanding of the author, infliximab was first described in 1993 as a chimeric human/mouse monoclonal anti-tumor necrosis factor alpha antibody (known as cA2) produced by Centocor Inc. (Malvern, Pennsylvania, United States) and experimentally used to treat rheumatoid arthritis (Elliott et al., 1993; Knight et al., 1993). Within the same year, it was used to experimentally treat a patient with severe Crohn’s disease with promising results (Derkx et al., 1993). Apart from consulting academic historians who are familiar with the historical development of infliximab, readers may also rely on review papers and meta-analyses to have an idea on the availability of randomized controlled trials published in the literature. In the current work, the method of cited reference analysis was demonstrated to identify the historical roots of infliximab research with Crohn’s disease, its first trial, and important references published prior to the first trial.

The pioneering works may provide insights to guide researchers for future studies. For instance, readers should appreciate Crohn et al. (1932) that reported a case series of patients affected by Crohn’s disease, described as regional ileitis at that time (Crohn et al., 1932). In order to classify patients into different groups according to disease severity for management triage, Best et al. (1976) developed the Crohn’s Disease Activity Index (CDAI) (Best et al., 1976). Also well-known by the research community are the ACCENT I and ACCENT II trials (Present et al., 1999; Hanauer et al., 2002). By identifying and studying these works in a chronological order from bibliometric data, researchers can better understand the development or evolution of treatment regimens with their corresponding patient groups. Several bibliometric studies concerning IBD have been published, including (but not limited to) analyses of the top 25 cited articles on COVID-19 and IBD (Veisman et al., 2022), immunotherapy and biotherapy for IBD (Xiong et al., 2022), original articles of the Crohn’s disease research literature (Karabulut and Kaya, 2023), and the use of ustekinumab for Crohn’s disease (Chen et al., 2024). However, none of them focused on infliximab. Besides, there is a lack of a historical overview of infliximab in Crohn’s disease management using bibliometric tools, which may offer another perspective apart from traditional review papers based on expert opinion. Hence, the current work aimed to demonstrate the use of cited reference analysis to identify and study the historical roots of research on the use of infliximab to treat Crohn’s disease. In addition, a disruption index for the most cited references identified from the analysis would be calculated. In short, a disruption index gives a score to a cited reference from −1 to +1, with −1 meaning a continuity from existing research and +1 meaning a disruption (Wu et al., 2019). Please the Materials and Methods section for more methodological details.

## 2 Materials and methods

The Web of Science Core Collection (WOSCC) literature database was queried on 14 August 2024 to identify studies on the use of infliximab to treat Crohn’s disease. The following search terms were used: (Infliximab OR Remicade OR Ixifi OR Renflexis OR Inflectra OR Remsima) AND (Crohn*). The former group of words were searched in the title, abstract, and author keyword fields of papers indexed in WOSCC, whereas the term Crohn* was searched in the title field only to make the dataset more specific. Papers were limited to those labelled as articles (original articles) by WOSCC. Besides this, no other restrictions were placed to limit the search. Finally, the search yielded 1,793 articles.

The full record and cited references of these articles were exported to CRExplorer (Thor et al., 2016) to undergo a method of cited reference analysis called reference publication year spectroscopy (RPYS). In short, the number of times that the cited references were cited by the 1793 articles were counted and sorted by the publication year of the cited references. The positive and negative peaks shown in an RPYS visualize years when the citation count of the cited references deviated from its 5-year median. Take the year 2002 when the ACCENT I trial was published as an example. From the downloaded dataset of 1793 articles, cited references published in 2000–2004 were cited 1,616, 1,647, 2,537, 2,187, and 2,580 times respectively. The 5-year median citation count was 2,187. It meant that references published in 2002 were cited 350 times more than its 5-year median and thus created a positive peak with a magnitude of 350. Many positive peaks were generated by RPYS. The original plan was to follow the routine of CRExplorer developers to identify “important peaks” with Tukey’s fences based on the interquartile range of the median deviations with positive values (Haunschild and Bornmann, 2022; Gruber et al., 2023). However, only one peak could survive the lower fence. Therefore, the final decision was to lower the threshold to the upper quartile. Peaks that had a magnitude exceeding the upper quartile were deemed significant in the current work. From each significant peak, the most cited reference was identified. Using CRExplorer, it was possible to identify reference publication years (RPY) that experienced a sudden increase in total citations received and to pinpoint the corresponding references responsible for the increase. This method enables users to identify important cited references that are frequently cited by a predefined literature set but may not have a very high total citation count to be recognized by traditional citation analysis.

Disruption index (DI) for the most cited references from the identified peaks were calculated. In short, a disruption index gives a score to a cited reference (also called a focal paper) from −1 to +1, with −1 meaning a continuity from existing research and +1 meaning a disruption (Wu et al., 2019). The formula considers the number of papers that cite exclusively the focal paper, exclusively the references of the focal paper, and both the focal paper and its references. There are many variants of disruption index in the literature, and the one used here is called DI_5_ proposed by Bornmann et al. (Bornmann et al., 2020). By obtaining data from WOSCC, DI_5_ of the most cited reference from the important peaks were computed with an automated routine described by Leydesdorff and Bornmann (Leydesdorff and Bornmann, 2021) and available from https://www.leydesdorff.net/software/di/. The computation of a disruption index could complement the findings from CRExplorer, allowing for further differentiation of the contextual nature of highly cited references.

## 3 Results

**FIGURE 1:**
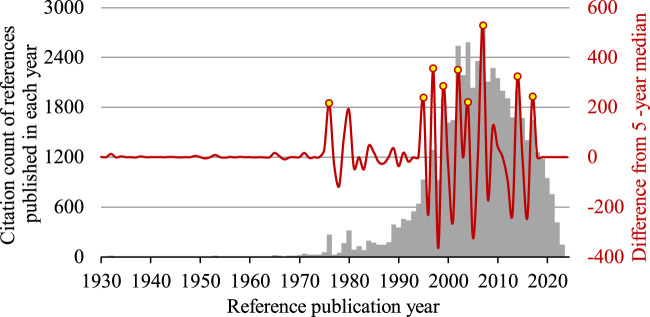
Reference publication year spectrogram of research on the use of infliximab to treat Crohn’s disease. Peaks with a magnitude that exceeded the upper quartile are identified in 9 years, namely 1976, 1995, 1997, 1999, 2002, 2004, 2007, 2014, 2017 (yellow). A very small peak is visible in 1932, when the seminal paper by Crohn et al. (1932) was published. The bar chart shows the citation count of references published in each reference publication year (grey), whereas the wave form shows the deviation of citation count from the 5-year median (red).

The 1,793 articles entered into the analysis had a total of 21,540 distinctive cited references published since 1900. Overall, there were 9 significant peaks identified between 1976 and 2017 (Figure 1). Table 1 shows a list of the most cited reference identified from each significant peak.

**TABLE 1:** Details of the most cited reference identified for each significant peak from reference publication year spectrogram shown in Figure 1.

Reference publication year (RPY)	Cited references	No. of citations from the 1,793 articles (overall WOSCC citation count)	% of citations received by all references published in the same RPY	Disruption index (DI_5_)
1976	Best et al. (1976)	178 (3,197)	66.2%	0.99
1995	Van Dullemen et al. (1995)	105 (964)	11.3%	0.10
1997	Targan et al. (1997)	426 (2,687)	33.0%	0.09
1999	Present et al. (1999)	412 (2091)	21.7%	0.10
2002	Hanauer et al. (2002)	574 (3,294)	22.6%	0.13
2004	Sands et al. (2004)	315 (1,616)	12.2%	0.01
2007	Colombel et al. (2007)	224 (1,686)	8.0%	−0.002
2014	Ruemmele et al. (2014)	50 (732)	2.5%	−0.03
2017	Gomollón et al. (2017)	72 (1,377)	4.4%	Not computed

The reference list of Gomollón et al. (2017) was placed in its supplementary data, and WOSCC, treated it as having 0 reference. Hence, its DI_5_ was not computed.

## 4 Discussion

This cited reference analysis has identified 9 important cited references from studies on the use of infliximab to treat Crohn’s disease.

The first important cited reference identified was Best et al. (1976). It reported the development of CDAI, an 8-factor psychometric scale used to evaluate the severity of the disease and developed based on prospective data collected by the National Cooperative Crohn’s Disease Study group on 112 patients. Best et al. (1976) also set threshold values of ≤150 for quiescent disease, and >450 for extremely severe disease. It only had three references, none of which was related to the disease activity of Crohn’s disease. In this sense, Best et al. (1976) was very innovative to collect patient data and construct a clinical assessment tool to classify Crohn’s disease patients according to disease severity. This was reflected by its high DI_5_ value of 0.99, almost approaching to 1. The development of CDAI has inspired the subsequent development of a simpler index known as the Harvey-Bradshaw index (HBI) (Harvey and Bradshaw, 1980), which does not require a patient to complete a diary card 1 week before assessment and does not impose any weighting factors.

The second important cited reference identified was van Dullemen et al. (1995). It was a case series of 10 patients with active Crohn’s disease (CDAI value > 200) who were unresponsive to ≥20 mg prednisone for ≥2 weeks. At Academic Medical Center, Amsterdam, Netherlands, an open-label treatment with a single intravenous infusion of cA2 (infliximab) at 10 mg/kg, administered over 2 h, resulted in significant clinical improvement in 8 of the 10 patients that generally lasted for 4 months. This case series explicitly mentioned itself as a follow-up study of a case report, Derkx et al. (1993), that treated one severe Crohn’s disease patient in the same hospital with the same treatment protocol. Even though Derkx et al. (1993) could be one of the first, if not exactly the first, to treat Crohn’s disease patient with infliximab, their case report did not produce a noticeable peak in the RPYS or have a high DI_5_ value (0.15), though its DI_5_ value was slightly higher than that of van Dullemen et al. (1995) (0.10).

The third important cited reference identified was Targan et al. (1997). It was recognized as the first-ever randomized controlled trial of infliximab in treating patients with Crohn’s disease (Sands, 2007; Hindryckx et al., 2014). A total of 108 patients with moderate-to-severe Crohn’s disease (CDAI value at 220–400) who were unresponsive to prior treatment were recruited from 18 centres in North America and Europe. In this double-blinded trial, patients were randomized into receiving a single 2-h intravenous infusion of placebo, or infliximab in the dose of either 5, 10, or 20 mg/kg. Results found that infliximab of 5 mg/kg had the best clinical response rate and remission rate compared to the other two dose regimens, and all of them were significantly better than placebo. Overall, the infliximab treatment was very effective at 4 weeks after the infusion, but patients showed signs of relapse at week 12 in terms of CDAI value and C-reactive protein concentration. The most common adverse effects of a single-dose infliximab infusion reported in this trial included headache, nausea, and upper respiratory tract infection; but the prevalence of these effects were comparable to the placebo group.

The fourth important cited reference identified was Present et al. (1999). Similar to Targan et al. (1997), it was also a double-blinded, placebo-controlled trial. This time, 94 Crohn’s disease patients with draining abdominal or perianal fistulas of >3 months were recruited from 12 centres in the United States and Europe. They were randomized into receiving 3 doses of either placebo, infliximab of 5 mg/kg, or infliximab of 10 mg/kg via intravenous infusion at weeks 0, 2 and 6. Results found that infliximab of both dose regimens had significantly better clinical response rate than placebo in terms of healing or complete absence of draining fistulas. Mean CDAI values of infliximab groups were also significantly lower than the placebo group at week 2, but not at week 18. In terms of adverse effects, the prevalence of headache seemed to be comparable across the placebo group and both infliximab groups, but the 10 mg/kg infliximab group tended to have a higher prevalence of having abscess, upper respiratory tract infection, and fatigue. Hence, Present et al. (1999) reaffirmed the initial dose of 5 mg/kg recommended by Targan et al. (1997), and further recommended subsequent identical doses at week 2 and week 6, thus forming the protocol of current intravenous induction regimen of using infliximab to treat Crohn’s disease patients.

The fifth important cited reference identified was Hanauer et al. (2002). It was a double-blind, placebo-controlled trial registered as the ACCENT I trial. A total of 573 Crohn’s disease patients (CDAI value ≥ 220) were recruited from 55 centres in North America, Europe, and Israel. The patients received an initial dose of 5 mg/kg intravenous infusion of infliximab at week 0, then randomized into receiving repeat infusions at weeks 2 and 6 of either placebo or infliximab in the dose of 5 mg/kg. For the placebo group, patients will then receive repeat infusions of placebo every 8 weeks until week 46. For the infliximab group, patients were further randomized into receiving repeat infusions of either 5 mg/kg or 10 mg/kg of infliximab every 8 weeks during the maintenance period, until week 46. Results found that patients who responded to the initial dose of infliximab were more likely to sustain clinical remission at weeks 30 and 54, discontinue the use of corticosteroid (prednisone, prednisolone, or budesonide), and maintain their response for a more prolonged period. The clinical remission and clinical response rates for the group with repeat infusions of 10 mg/kg of infliximab during the maintenance period were better than the group with 5 mg/kg, but the differences did not reach statistical significance. In fact, Hanauer et al. (2002) commented in its introduction that the maintenance regimen of infliximab infusion every 8 weeks was previously tested by their study group, published as Rutgeerts et al. (1999). In the study by Rutgeerts et al. (1999), survival analysis for time to loss of response showed that the median time to loss of response for the infliximab retreatment group and the placebo group were 48 weeks and 37 weeks, respectively, with a P-value of 0.057. Hanauer et al. (2002) commented that the results of Rutgeerts et al. (1999) were promising but underpowered, and therefore designed a larger study to confirm the efficacy. As such, they established the protocol of current maintenance regimen of repeat infusions every 8 weeks.

The sixth important cited reference identified was Sands et al. (2004). It was a double-blind, placebo-controlled trial registered as the ACCENT II trial. A total of 306 Crohn’s disease patients with draining abdominal or perianal fistulas of >3 months were recruited from 45 centres in North America, Europe, and Israel. The patients received the induction regimen of 5 mg/kg intravenous infusion of infliximab at weeks 0, 2, and 6. A total of 282 patients entered randomization at week 14 to receive the maintenance regimen of 5 mg/kg infliximab or placebo every 8 weeks until week 54. Results found that the time to loss of response was significantly longer for patients in the infliximab group than those in the placebo group, and the infliximab group had a significantly higher ratio of patients with a complete absence of draining fistulas than the placebo group at week 54. This trial can be perceived as a follow up study of both Present et al. (1999) and Hanauer et al. (2002) to demonstrate that the recommended maintenance regimen can be equally applicable to patients with draining fistulas.

The seventh important cited reference identified was Colombel et al. (2007). It was a double-blind, placebo-controlled trial registered as the CHARM trial. As stated in its introduction, the CHARM trial was conducted because previous findings showed that repeat infusions of infliximab would lead to the development of antibodies, rendering loss of efficacy, infusion reactions, and delayed hypersensitivity. Hence, Colombel et al. tried to test adalimumab, another biologics with a similar function. Results found that it was significantly better than placebo in maintaining remission in moderate-to-severe Crohn’s disease through 56 weeks. They concluded that adalimumab could be a substitute of infliximab for patients who were intolerant of or failed to respond to the latter.

From van Dullemen et al. (1995) to Colombel et al. (2007), several authors have been involved in 3 or more studies, namely Paul Rutgeerts (n = 5), Stephen B. Hanauer (n = 4), and Sander J. van Deventer (n = 3), as well as many authors who had contributions to 2 studies, such as Jean–Frédéric Colombel, Daniel H. Present, and Bruce E. Sands, to name a few. It showed that many highly cited references identified in this study were authored by a core group of field experts in North America and Europe.

The eighth and ninth important cited references identified were Ruemmele et al. (2014) and Gomollon et al. (2017). These two papers were European consensus guidelines on the management of pediatric Crohn’s disease and the diagnosis and management of Crohn’s disease, respectively. Both papers were initiatives from the European Crohn’s and Colitis Organisation (ECCO).

Meanwhile, it was worthwhile to mention that the first visually noticeable peak, being very small and not reaching the threshold set to identify important peaks, was found in 1932 by RPYS. The most cited reference published in that year was Crohn et al. (1932) that reported a case series of 14 patients with regional ileitis, now known as Crohn’s disease (14 citations, or 93.3% of citations received by references published in 1932).

Undoubtedly, there were many clinical trials on the efficacy of biologics (either infliximab or others) in treating Crohn’s disease patients. For a comprehensive (but not exhaustive) list of the trials, readers can refer to recent reviews and meta-analyses (Hindryckx et al., 2014; Singh et al., 2021; Barberio et al., 2023; Shehab et al., 2023). Meanwhile, the current study has demonstrated the ability of cited reference analysis to identify seminal papers from a pre-defined literature set, such as papers that reported the invention of CDAI, the very first randomized controlled trial of infliximab on Crohn’s disease, and the renowned ACCENT I and II trials. This method of cited reference analysis offers a different perspective than traditional citation analysis. Traditionally, citation analysis focused on the total number of citations received by papers. However, some papers are only important in a small research field. Even if they are frequently cited by papers within the small research field, their overall citation count will still be very low, so that they may not be readily identified by a routine search. Moreover, the historical insights could help inform new research areas or gaps in Crohn’s disease treatment. For example, depending on the pharmacological similarity, newer biologics may be tested with similar induction and maintenance regimens. In particular, since repeated infusions of infliximab would result in loss of efficacy, infusion reactions, and delayed hypersensitivity due to the development of antibodies, newer biologics should be tested in these aspects, and have a much more delayed or minimal level of antibody development.

This study had several limitations. First, the accuracy of cited reference and citation count depended on the literature database. It was reasonable to expect that literature databases, such as WOSCC used in this study, may fail to keep a record of the reference list of very old publications, or even fail to index older publications themselves (Yeung, 2023). However, the current study has revealed that even recent publications indexed in WOSCC may suffer from the same issue, as in the example of Gomollón et al. (2017) that placed its reference list in its supplementary data, rendering WOSCC recording it as having 0 reference. Moreover, well-established concepts and treatment regimens may become a common knowledge that the source articles are no longer cited, otherwise known as obliteration by incorporation (Merton, 1965; Yeung, 2021). On the other hand, the calculation of the disruption index makes use of bibliographic data only. Therefore, even though some of the cited references were definitely clinically novel, such as the first attempt to use infliximab to treat Crohn’s disease patients or the first randomized controlled trial on the efficacy of infliximab to treat Crohn’s disease patients, they were considered not disruptive from the perspective of bibliometric data. These issues might underestimate the scientific impact of the analyzed papers.

In conclusion, this cited reference analysis on the use of infliximab to treat Crohn’s disease succeeded in identifying key references of the literature. The first important reference identified within the analyzed dataset was Best et al. (1976), which introduced the CDAI to assess the disease severity of patients. Key case series and randomized controlled trials, as well as consensus guidelines, were also identified. It is anticipated that randomized controlled trials that compare the efficacy between infliximab and other newer biologics in managing Crohn’s disease in various stages will become highly cited in the future. Therefore, future bibliometric studies can explore and reveal the research foundation of newer biologics and targeted medicines used to treat IBD, such as interleukin inhibitors, integrin blockers, sphingosine-1-phosphate receptor modulators, and Janus kinase inhibitors.

## Data Availability

The original contributions presented in the study are included in the article/supplementary material, further inquiries can be directed to the corresponding author.
